# Cumulative life course adversity, mental health, and cognition in the UK biobank

**DOI:** 10.1038/s41598-022-18928-9

**Published:** 2022-08-29

**Authors:** M. Künzi, D. A. Gheorghe, M. Kliegel, N. Ballhausen, J. Gallacher, S. Bauermeister

**Affiliations:** 1 Cognitive Aging Lab, Faculty of Psychology and Educational Sciences, University of Geneva, Boulevard du Pont d’Arve 28, 1205 Geneva, Switzerland; 2 Center for the Interdisciplinary Study of Gerontology and Vulnerability, University of Geneva, Geneva, Switzerland; 3 LIVES, Overcoming Vulnerability: Life Course Perspective, Swiss National Centre of Competence in Research, Lausanne, Geneva, Switzerland; 4 Department of Experimental and Theoretical Neuroscience, Transylvanian Institute of Neuroscience, Cluj-Napoca, Romania; 5 Dementias Platform UK, Department of Psychiatry, University of Oxford, Oxford, UK; 6 Department of Developmental Psychology, Tilburg School of Social and Behavioral Sciences, Tilburg University, Tilburg, The Netherlands

**Keywords:** Psychology, Human behaviour

## Abstract

The association between adversity and cognition varies according to the specific adversity, when the adversity was experienced, and the cognitive domains investigated. Disentangling the effect of adversity and the underlying mechanistic pathway is therefore difficult. The association between adversity (i.e., maltreatment) accumulated over the life course and cognitive flexibility, as well as two potential mediators (i.e., intra-individual variability in reaction time and depression) of this association, were investigated. Data stem from the baseline population of the UK Biobank study (*N* = 73,489, *Mdn*_*age*_ = 56, *SD*_*age*_ = 7.628, 55.740% of women). Cumulative life course adversity (specifically maltreatment) was measured with items based on the Childhood Trauma Questionnaire (CTS-5) and items adapted from the British Crime Survey. Depression was assessed with the Patient Health Questionnaire-9 (PHQ-9). Intra-individual variability in reaction time was measured with a reaction time test “snap game” and the Trail Making Test A and B were used as a measure of cognitive flexibility. A path analysis was performed on these data. Higher cumulative adverse experiences were associated with lower performance in cognitive flexibility (β = .016, *p* < .001, 95% CI [0.009, 0.024]), and this effect was partly mediated by the level of depression (22.727% of the total effect of cumulative life course adversity on cognitive flexibility was mediated by depression (β = .005, *p* < .001, 95% CI [0.004, 0.007])). No association between cumulative life course adverse experiences and intra-individual variability in reaction time was found, nor was any indirect association between cumulative life course adversity and performance in cognitive flexibility via intra-individual variability in reaction time. The association between cumulative life course adversity, depression, and performance in cognitive flexibility has been highlighted. In contrast, no indirect effect between cumulative life course adversity and performance in cognitive flexibility via intra-individual variability in reaction time was found, suggesting that it is not a potential mechanism underlying the association between cumulative life course adversity and executive function.

## Introduction

Existing literature on the associations between adversity and cognition later in life has reported both a negative^1–4^ and a positive relationship^5–7^. Given the diversity of adverse experiences ranging from everyday life conditions to traumas^8–10^, these findings highlight that further factors should be considered when exploring the relationship between adversity and cognition. Specifically, existing results on the association between adversity and brain and/or cognition vary according to the specificity (severity), number, duration as well as the chronicity of adversity, the life course period at which the adversity was experienced, and the cognitive domains under investigation^6,8,11^. Moreover, it is considered to be difficult to disentangle the effect of adversity since different adversities frequently occur simultaneously^12^, adversities experienced at one stage of life are often associated with adversities experienced in the subsequent stage^2^, early life events influence brain response to adulthood stressors^13^, and accumulated adversity may foster resilience^14^. Therefore, it is important to investigate the effect of adversity accumulated over the life course (i.e., cumulative adversity), and precisely to avoid mixing types of adversity and finding contradictory results; it would be advantageous to avoid confounding the possibly contrary effects of different adversity types by investigating only one specific type of adversity accumulated over time. Moreover, to better understand the association between one specific adversity cumulated over the life course and cognition, it is also important to define the mechanisms underlying this relationship.

### Cumulative adversity

Within the context of adversity, it is important to consider stress theory and how individual experiences in early and adult life shape the response to stress^13^. Stressful experiences may lead to structural and functional changes in the brain and under certain conditions may impair mental and physical health^15^. Too much stress that may be caused by repeated hits from multiple stressors or by a chronic stressor, results in wear and tear on the body and brain (allostatic load or overload)^13,15^. More specifically, repeated stress has been shown to be associated with functional and structural changes (e.g., dendritic remodeling, shortening, and growth) in different brain regions (hippocampus, cortex prefrontal, and amygdala)^13,16^. For example, accumulation of adverse experiences is associated with lower gray matter volume in key regions of the brain involved in emotion regulation, contextual processing, and self-control^17^. Furthermore, this smaller gray matter volume in specific brain regions (medial prefrontal cortex) in individuals with a history of more cumulative adverse life events has been hypothesized as potentially partially playing a role in the increased risk for depression, addiction, and other stress-related psychopathology^17^. Interestingly, an association between the accumulation of adverse experiences and the increase risk of depressive disorders as well as lower cognitive performance, has also been reported^18,19^. However, it has been suggested that rather than the ‘accumulation of adversities’, it is the ‘dosing’ of adversity experienced (i.e., neither none nor too much but some adversity experiences) that is important. Indeed, the ‘dosing’ of adversity experienced seems to play a key role in the relationship between adversity and mental health, as well as well-being outcomes^14^. Specifically, a moderate level of adversity experienced may be important to foster resilience and lead to better mental health and well-being outcomes compared to individuals with no experience of adverse experience and too many adverse experiences^14^. A further study suggests that the severity of each adverse experience should also be considered in the study of cumulative adversity. Indeed, the association found between young adults who have experienced higher cumulative adverse events and poor emotional and behavioral functioning seems to be due to both the number and the severity of the adverse events experienced^20^.

In the present study, to avoid mixing (types of) adversities and differential impacts on investigated outcomes (e.g., low-impact adversities with high-impact adversities) and therefore underestimating the effect of high-impact adversities, it was decided to investigate the association between severe adversities belonging to the same category (i.e., maltreatment) accumulated over the life course. Importantly, this study is based on the maltreatment definition which includes physical, sexual, emotional, financial abuse and neglect^10^.

### Mechanisms

The mechanistic pathways underlying the links between adversity and cognition are yet to be understood, however, one of the most commonly cited adversity- or stress-induced effects are thought to be structural brain changes (e.g., Lupien et al^21^). Stress or adversity experienced in a sensitive period of life (childhood and/or late-life) has been shown to lead to long-lasting effects on brain structures, impacting cognition^4,21^. Specifically, adversity or stress experienced in childhood and adolescence may affect the development of the brain. The prefrontal cortex (PFC), due to its late maturation, has been highlighted as being a brain region particularly vulnerable to the effects of stress and particularly severe stressors such as maltreatment^21,22^. Importantly this brain region is important for specific cognitive functions, such as executive functions^23,24^. The experience of adversity (specifically the experience of childhood maltreatment) has also been associated with white matter integrity^8,11,23,25^. White matter integrity in turn is linked to processing speed and cognitive performance such as executive functioning^26^. Of importance is that, since alterations in white matter integrity affect processing speed, intra-individual variability in reaction time (RT) is also thus a sensitive indicator of white matter integrity^27^, and further, it is also associated with impairment and decline in cognitive performance^28,29^. These works suggest a tentative mechanistic relationship between the experience of adversity, the development of the PFC, the disruption of white matter integrity, an increase in intra-individual variability in RT, and lower performance in cognitive tasks, especially, tasks assessing executive functioning.

In particular and interestingly, maltreatment (i.e., childhood maltreatment) has been reported to affect memory, working memory, attention, and motor inhibition^22^. In parallel with these cognitive performance findings, the brain regions shown to be consistently impacted by childhood maltreatment are the PFC, and the anterior cingulate cortex (ACC). Furthermore, prefrontal white matter microstructure has been found to be affected in children that experienced early neglect, which may be linked to the deficit in cognition associated with early neglect^25^. Moreover, children who suffered from early neglect have lower total white matter volume compared to children who do not^25^. In terms of cognitive performances, cumulative childhood maltreatment has been shown to be related to lower executive functioning in adulthood^19^. These studies show how vulnerable the PFC and the white matter microstructure are to the experience of early maltreatment. Hence, maltreatment may be associated with lower performance in executive functions due to the disruption of the development of the PFC, especially white matter integrity which may subsequently be reflected in intra-individual variability in RT performance values.

A second mechanism that seems to play a key role in explaining the relationship between adversity and cognition is depression. Depression is adversely affected by adversity and stress, and adversely affects cognitive performance, as outlined in the following findings. A strongly graded relationship between the total number of cumulative adverse childhood experiences and recent, as well as lifetime history of depression, has been found^18,30^. This finding suggests that childhood adversity has mental health consequences extending far into adulthood. Interestingly, emotional abuse, a form of maltreatment, has been found to have a stronger relationship with depression compared to other childhood adverse experiences^18,30^. In line with this finding, a stronger positive association between emotional abuse and neglect (further specific experiences of maltreatment) with depression has been reported^31^. Although it is important to note that a positive association between each type of childhood maltreatment and depression has also been found (i.e., physical abuse, sexual abuse, and physical neglect)^31^.

To determine whether depression may be a potential explanatory pathway by which adversity and specifically early maltreatment influences cognition (i.e., executive functions), a link between depression and cognition warrants investigation. An association between patients currently depressed or in remitted states and cognitive impairment (e.g., executive function and attention) is well established^32,33^, with an increase in depression severity associated with cognitive impairment in executive function, episodic memory, and processing speed^34^. One suggestion is that depression-related cognitive impairment may be due to hyperactivity of the hypothalamic–pituitary–adrenal (HPA) axis leading to the elevated production of cortisol which in turn leads to the cognitive impairments^35,36^.

Hence, these various studies suggest an important relationship between adversity, depression, and cognition, particularly executive functioning, potentially through the HPA axis. Thus, it may be plausible that adversity, specifically maltreatment, might lead to depression, which in turn could affect cognition (i.e., executive functioning), and that depression may explain the relationship between adversity and cognition.

The aim of the present study is to investigate the association between experiences of maltreatment accumulated over the life course and cognitive performance by performing a path analysis on the baseline population of the UK Biobank population-based prospective cohort study. In light of the relationships highlighted in the literature between maltreatment, intra-individual variability in RT, depression, and executive functioning, this study aims to investigate whether intra-individual variability in RT and depression are the mediators of the association between cumulative experiences of maltreatment over the life course and performance in cognitive flexibility (executive function). We predicted that (1) cumulative experiences of maltreatment over the life course predict lower performance in cognitive flexibility (i.e., direct effect), (2) cumulative experiences of maltreatment over the life course predict both higher intra-individual variability in RT and depressive symptoms (i.e., direct effects), (3) both higher intra-individual variability in RT and depressive symptoms predict lower performance in cognitive flexibility (i.e., direct effects), and (4) both intra-individual variability in RT and depressive symptoms mediate the relationship between cumulative experiences of maltreatment over the life course and performance in cognitive flexibility (i.e., indirect effects).

## Methods

### Participants

The study included the baseline of the UK Biobank, a large population-based prospective cohort study which was established in 2006 to investigate both genetic and non-genetic diseases of middle and older aged adults. Participants were recruited across the United Kingdom through the UK National Health Service (NHS) record system. Comprehensive sociodemographic, lifestyle, medical history, environmental, cognitive, and biomedical data on 502,655 participants aged 37 to 73 years was collected through touchscreen assessments and physical examination^37,38^. Additional questions on mental health (depression and manic symptoms) were included during the last two years of the baseline assessment (therefore information is not available for all participants)^39^. All cognitive tasks were performed unsupervised on a touchscreen and have shown to be correlated with general cognitive ability and test–retest reliability^40^. UK Biobank study received ethical approval from the Research Ethics Committee (approval letter dated 17th June 2011, Ref 11/NW/0382) and was conducted in accordance with the Declaration of Helsinki. All participants gave written informed consent for their participation. Only participants without missing values in the variables and items used to create the variables included in the model (i.e., cumulative life course adversity, depression, Trail Making Test ratio, age, gender, and education) except intra-individual variability in RT (for which multiple rounds were available) were used in the analysis (*N* = 73,489, *Mdn*_*age*_ = 56, *SD*_*age*_ = 7.628, 55.740% of the participants were women; see Figure A in the supplements for participants selection).

## Materials

### Adversity

Items representing adversity were selected based on the existing definitions of adversity (i.e., highly stressful, and potentially traumatic, events or situations), whilst also focusing on the specific concept of maltreatment experienced in childhood as well as in adulthood^10,41^. The items assessing childhood adversity (i.e., I felt that someone in my family hated me; I felt loved a child; People in my family hit me so hard that it left me with bruises or marks; There was someone to take me to the doctor if I needed it) were based on the Childhood Trauma Questionnaire (CTS-5)^42^, and those assessing adulthood adversity (i.e., A partner or ex-partner repeatedly belittled me to the extent that I felt worthless; A partner or ex-partner deliberately hit me or used violence in any other way) were adapted from the British Crime Survey^43^. The items were dichotomized and recoded as 0 = never having experienced the adverse event, and 1 = having experienced the adverse event, following the same procedure as Gheorghe et al^41^. In addition to the dichotomizing and recoding of the items, a cumulative score of adversity was computed by summing up the four childhood adversity items and the two adulthood adversity items. This cumulative life course adversity score ranges from 0 = no adversity experienced up to 6 = all adversities experienced (see Table 1).

**Table 1:** Frequency table of the cumulative adversity over the life course.

Cumulative adversity over the life course	Frequency	Percent	Cumulative percent
0	41,112	55.943	55.943
1	17,626	23.985	79.928
2	9,569	13.021	92.949
3	3,595	4.892	97.840
4	1,390	1.891	99.732
5	183	0.249	99.981
6	14	0.019	100
Total	73,489	100	

### Intra-individual variability in RT

To measure intra-individual variability in RT, a RT test “snap game” was performed (https://biobank.ctsu.ox.ac.uk/crystal/crystal/docs/Snap.pdf). Precisely, the item “*Duration to first press of snap-button in each round”* was used for each of the rounds (0 to 11). This item is a measure of the time interval (ms) between the display of the two cards and the button press made by the participant to indicate a match between the two symbols of the two displayed cards. Note that this item does not differentiate between proven matches and mistakes. Following the procedure of Haynes et al^28^, a coefficient of variation (CV) was calculated by dividing the intra-individual standard deviation in RT by the intra-individual mean RT. This CV has the advantage to control for the individual mean RT producing a metric which reflects variability rather than processing speed^44^. Training rounds (rounds 0–4), as well as RT under 50 ms (anticipation) and over 2000 ms (timing at which the cards had disappeared), were excluded (for the same procedure, see Piumatti et al^45^). The CV was only calculated for participants having at least four rounds without missing data. Note that none of the participants had a RT greater than 3 standard deviations that required to be excluded.

### Depression

Following the procedure of Bauermeister and Gallacher^37^, an Item Response Theory (IRT) graded response model for Likert-type items was fitted to the 9 items of the Patient Health Questionnaire-9 questions (PHQ-9; coded from 0 not at all to 3 nearly every day to the question: “how often have you been bothered by any of the following problems”). The aim of performing an IRT analysis on the items assessing depression is to obtain a prediction of the individual θ scores on the fitted IRT model that will be used as a latent construct in the Structural Equation Models (SEM), an alternative to the summed scores. The IRT has the great advantage of avoiding important limitations raised regarding summed scores (e.g., equality of differences in the Likert scale but also equality of item weights in a scale^46^).

This IRT analysis suggested that the 9 items of the Patient Health Questionnaire-9 questions (PHQ-9) measure only the absence or presence of depression (high levels of depression) and do not differentiate people with absent or low (4, 3, 2, and 1) levels of depression. The questions are mostly differentiating individuals with high levels of depression (1, 2, and 3). Although less information is reliable for individuals with average (0) and extreme (4) levels of depression, this is still acceptable^37^ (Table 2).

**Table 2:** Reliability for values of Ө from a graded response model fit for the Patient Health Questionnaire-9 questions (PHQ-9).

Ө	TIF	TIF SE	Reliability
− 4	1.006	0.997	0.006
− 3	1.030	0.985	0.029
− 2	1.162	0.928	0.139
− 1	1.890	0.727	0.471
0	4.870	0.453	0.795
1	15.840	0.251	0.937
2	20.423	0.221	0.951
3	10.956	0.302	0.909
4	3.431	0.540	0.709

*TIF* Test Information Function, *SE* Standard error.

A second IRT model was fitted by removing the item (“Moving or speaking so slowly that other people could have noticed? Or the opposite—being so fidgety or restless that you have been moving around a lot more than usual”) with a discriminant value lower than 1.27. Since no discriminant value was lower than 1.7, no more items were removed from the scale, and a prediction of the individual θ scores on the fitted IRT model was computed only for the participant without missing values.

### Cognition

The Trail Making Test (TMT) A and B were used as a measure of executive functioning^47^. Part A (TMTA) is the time in seconds to correctly connect the numbers 1 to 25 in ascending order. Part B (TMTB) is the time in seconds to correctly connect the numbers 1 to 13 and the letters A to L in ascending and alphabetic order alternating between number and letter (1-A, 2-B, 3-C,…,12-L-13). To keep only the flexibility component (executive function) a ratio score was calculated by dividing the TMTB by the TMTA^48^.

### Covariates

Age, gender (coded 0 for women and 1 for men), and education, item named qualifications in the UK Biobank (coded 1 = postgraduate degree, 2 = college or university degree, and 3 = secondary degree; see Bauermeister et al^49^), were used as covariates.

### Statistical analyses

**Figure 1:**
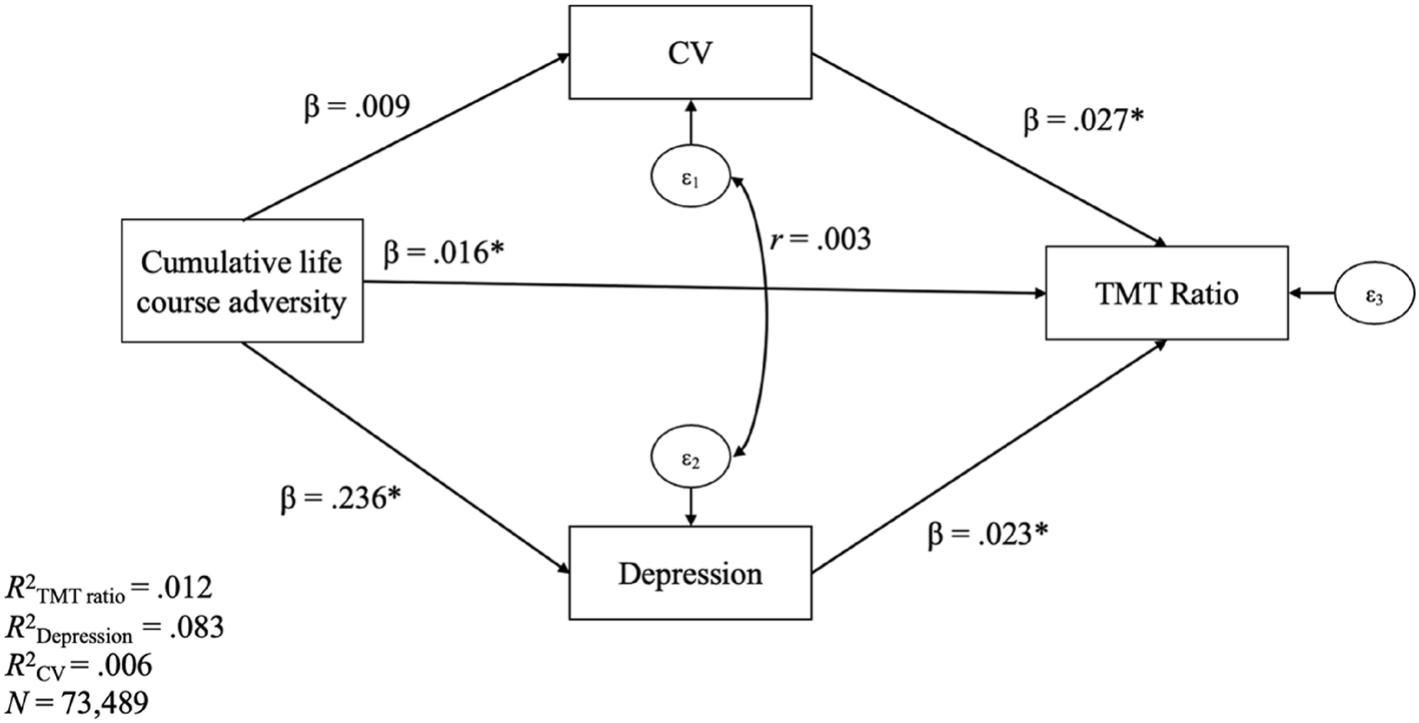
Standardized coefficients for the path analysis and explained variance for each endogenous variable of the model. *p < .001, CV = Coefficient of variation, *TMT* The Trail Making Test. *Note.* For clarity, pathways with the covariates are not reported.

The analyses were performed with STATA V.16.1 (StataCorp, College Station, TX, USA). First, Spearman correlations with a Bonferroni correction were computed, then a path analysis including all covariates was performed (α  =  0.010 to limit type I error given de large sample size^50^). To avoid the problem of the non-normality of the data, the asymptotic distribution-free (ADF) estimation method was used. The links in the path analysis model were defined such as the TMT ratio was predicted by cumulative life course adversity, CV, and depression. Depression and CV were predicted by cumulative life course adversity. Given the lack of consensus in the literature regarding the direction of the relationship between CV (used as an indicator of the white matter integrity in the study) and depression^51–54^, it was decided to correlate the errors of CV and depression. Regarding the covariates, gender and age predicted depression and TMT ratio, and age also predicted CV, finally, education only predicted TMT ratio (see Fig. 1 for a simplified illustration of the model tested). The model’s goodness of fit was assessed using the comparative Fit Index (CFI), considered as good when it is equal to or higher than 0.95, the Root Mean Squared Error of Approximation (RMSEA), considered as good when lower than 0.06, and the Standardized root-mean-square residual (SRMR), considered as good when equal to or lower than 0.08^55,56^.

## Results

Table 3 shows the Spearman correlations with a Bonferroni correction between all the variables entered in the model including covariates. Higher cumulative life course adversity was significantly correlated with a higher level of depression, being younger, female, and having a lower education level. Higher intra-individual variability in RT was significantly correlated with lower performance in cognitive flexibility, being older, female, and having a lower education level. A higher level of depression was significantly correlated with being younger age, female, and having a lower education level. Lower performance in cognitive flexibility was correlated with older age, being male, and a lower education level.

**Table 3:** Descriptive statistics and Spearman correlations with a Bonferroni correction for all the variables of the model.

Variable	*M*	*Mdn*	*SD*	Minimum	Maximum	1	2	3	4	5	6	7
1. Cumulative life course adversity	0.736	0	1.015	0	6	1.000						
2. CV	0.134	0.119	0.078	0	0.945	− .003	1.000					
3. Depression	− 0.024	− 0.104	0.821	− 0.799	3.490	.235**	− .007	1.000				
4. TMT ratio	1.745	1.670	0.501	0.248	16.218	.005	.032**	.011	1.000			
5. Age	55.531	56	7.628	38	72	− .119**	.074**	− .146**	.087**	1.000		
6. Gender						− .111**	− .023**	− .077**	.035**	.100**	1.000	
7. Education	2.383	2	0.580	1	3	.017**	.013*	.035**	. 050**	− .007	− .009	1.000

*p ≤ .01. **p ≤ .001.

*CV* Coefficient of variation, *TMT* The Trail Making Test.

For the path analysis, the fit of the model was very good, CFI = 0.978, RMSEA = 0.025 and SRMR = 0.008. Figure 1 shows the relationships between the variables entered in the model. Note that for clarity, the covariates were not drawn. There was no multicollinearity between the exogenous variables of the model (i.e., cumulative life course adversity, age, gender, and education) as indicated by the variance inflation factor (VIF) values equal or lower than 1.03. Higher cumulative life course adversity significantly predicted a higher level of depression and lower performance in cognitive flexibility but did not predict intra-individual variability in RT. Higher intra-individual variability in RT and a higher level of depression significantly predicted lower performance in cognitive flexibility (see Table 4). No correlation between the errors of intra-individual variability in RT and depression was found (*r* = 0.003, *p* = 0.365, 95% CI [– .004, 0.011]).

**Table 4:** Standardized coefficients of the model, with *z*, *p*-value, and associated 95% confidence interval.

Dependent variables	Predictors	Standardized coefficients	*z*	*p*-value	95% confidence interval	
					Lower limit	Upper limit
**TMT ratio**						
	Cumulative life course adversity	.016	4.18	< .001	.009	.024
	CV	.027	7.02	< .001	.019	.034
	Depression	.023	6.02	< .001	.016	.031
	Age	.090	24.13	< .001	.083	.098
	Gender	.019	5.04	< .001	.011	.026
	Education	.045	11.95	< .001	.038	.053
**CV**						
	Cumulative life course adversity	.009	2.36	.018	.001	.016
	Age	.075	20.68	< .001	.068	.082
**Depression**						
	Cumulative life course adversity	.236	61.81	< .001	.229	.244
	Age	– .126	– 35.25	< .001	– .133	– .119
	Gender	– .035	– 9.67	< .001	– .041	– .028

*CV* Coefficient of variation, *TMT* Trail Making Test.

The indirect effect of cumulative life course adversity on performance in cognitive flexibility mediated via depression was significant, i.e., higher cumulative life course adversity was associated with lower performance in cognitive flexibility, and this effect was partly mediated by higher level of depression (see Table 5). Precisely, 22.727% of the total effect of cumulative life course adversity on performance in cognitive flexibility was mediated by depression. The indirect effect of cumulative life course adversity on performance in cognitive flexibility via intra-individual variability in RT was not significant.

**Table 5:** Standardized coefficients of the indirect effect of cumulative life course adversity on TMT ratio via CV and depression.

Mediator	Standardized coefficients	*z*	*p*-value	95% confidence interval	
				Lower limit	Upper limit
CV	.001	2.23	.026	.001	.001
Depression	.005	5.98	< .001	.004	.007

*CV* Coefficient of variation.

For the covariates, older individuals showed higher intraindividual variability, a lower level of depression, and lower performance in cognitive flexibility. Being male was associated with a lower level of depression and lower performance in cognitive flexibility, and having a higher education level was associated with better performance in cognitive flexibility (see Table 4).

### Secondary analyses

To determine whether our results were specific to the cumulative adversity over the life course, we used the same model but replaced cumulative adversity over the life course with cumulative adversity experienced in childhood (model 1) and cumulative adversity experienced in adulthood (model 2) and added both cumulative adversity experienced in childhood and adulthood in the same model (model 3). The results are available in the supplements (see Tables A to F).

## Discussion

The present study investigates the association between cumulative adversity (i.e., maltreatment) over the life course and performance in cognitive flexibility, and two potential mechanisms (i.e., intra-individual variability in RT and depression) that may underlie the understanding of this association. The results show that higher cumulative life course adversity (i.e., maltreatment) is associated with lower performance in cognitive flexibility and this effect is partly mediated by the level of depression. This finding is in line with the literature showing a negative association between the experience of (cumulative) adversity and cognitive performance^1–4,19,22,25^. Although cumulative life course adversity (i.e., maltreatment) has a direct effect on the performance in cognitive flexibility, this result is partly mediated by the level of depression. The results found are in accordance with the literature showing an association between (cumulative) adversity and depression^9,18,30,31,57^ as well as the association between depression and lower cognitive performance mainly in executive functioning^32–34^. Hence our results support the hypothesis of a direct association between cumulative experiences of maltreatment over the life course and performance in cognitive flexibility, but also suggest that depressive symptoms partially mediate this association.

However, although our results exploring the association between higher intra-individual variability in RT and lower cognitive performance replicated those found in the literature^28,29^, the association between cumulative life course adversity (i.e., maltreatment) and performance in cognitive flexibility is not explained by the intra-individual variability in RT, a potential indicator of white matter integrity. Contrary to the association between adversity (specifically the experience of childhood maltreatment) and white matter integrity shown in the literature^8,11,23,25^, cumulative life course adversity (i.e., maltreatment) was not associated with intra-individual variability in RT in the present analysis. As existing research suggests that stress or adversity affects the brain and hence cognition mostly during childhood or late-life^4,21^, it might be possible that in this study, the effect of adversity experienced in childhood is masked by the adversity experienced in adulthood, as the adversities are cumulated over the life course and not separated according to the life course stages. To test this hypothesis, we used the same model but replaced cumulative adversity over the life course with cumulative adversity experienced in childhood and cumulative adversity experienced in adulthood, and added both cumulative adversity experienced in childhood and adulthood in the same model. Our results did not support this hypothesis and suggested that only cumulative adversity experienced in adulthood was associated with intra-individual variability in RT and by implication, white matter integrity. Importantly to note, this association disappeared when cumulative childhood adversity was included in the model. An explanation of this unexpected result may be that intra-individual variability in RT is not an optimal indicator of white matter integrity in this sample. Interestingly, the results of this third model show that only cumulative childhood adversity significantly predicted performance in cognitive flexibility, although an indirect effect via depression for both, cumulative childhood adversity and cumulative adulthood adversity, was significant. This result suggests that the direct effect of cumulative adverse experiences over the life course on performance in cognitive flexibility might stem from cumulative childhood adversity (see Figure B in the supplements for a visual summary of the significant paths found in the models tested). However, further studies are needed to support this finding, as only two items were used for the cumulative adversity score in adulthood.

In line with the literature findings showing an association between aging and decline in executive functioning performance and between aging and higher intra-individual variability in RT, being older was found to be associated with lower performance in cognitive flexibility, and higher intra-individual variability in RT ^28,58–61^. Being older was associated with a lower level of depression, the results found, given the age of the sample size, seem to be consistent with the literature findings^62,63^. Being female was associated with better performance in cognitive flexibility and a higher level of depression, supporting previous results showing gender differences in cognition as well as depression^62,64–66^. Finally, having achieved a higher educational qualification has been associated with better performance in cognitive flexibility, which is in line with the findings associating a higher level of education with higher levels of cognitive function^67^.

### Strengths and limitations

The present study investigated the associations between cumulative experiences of maltreatment over the life course, intra-individual variability in RT, depression, and a component of executive functioning (i.e., flexibility) using a large sample size. This study has the advantage to focus on one type of severe adversity (i.e., maltreatment) accumulated over the life course, therefore, avoiding mixing types of adversity of different impacts which would underestimate the effect of high impact adversities^20^.

In terms of limitations, this study assessed adversity retrospectively which may lead to recall biases (which could explain the higher cumulative life course adversity reported in younger adults), although good test–retest of the adverse childhood experiences have been previously demonstrated^68^. Moreover, the onset of depressive symptoms may be prior to the adverse experiences, and the influence of participants' level of depression on the reporting of adverse experiences cannot be excluded. Moreover, intra-individual variability in RT may be a better indicator of white matter integrity whether mismatches are accounted for, which was not possible with this data. Finally, it cannot be excluded that individuals participating in this study are in better cognitive and mental health. Possibly, individuals reporting adverse experiences in this sample may be particularly resilient, while individuals that are not resilient did not participate in the study or did not answer questions related to adversities. Importantly, however, this potential selection bias would lead to an underestimation of the results found.

### Outlook

To determine if the results found are cohort-specific and to better understand the results found, future research should replicate the findings of this study to other cohorts. Moreover, to have direct access to the brain, diffusion tensor imaging (DTI) based measures of white matter integrity in addition to intra-individual variability in RT should be included.

Our results and those of Ansell et al^17^ shed light on a potential new interesting association that future studies should investigate: the association between cumulative adversity, gray matter volumes, depression, and cognition. Furthermore, as the experience of maltreatment in childhood has been related to functional brain differences^22^, it would be interesting to examine the associations between childhood maltreatment experiences with structural brain changes and functional brain differences in relation to cognitive performance. Finally, our approach should be adapted and extended to other adverse experiences categories and cognitive outcomes to determine what are the mechanisms in play. For example, another cognitive ability that might be of particular interest to investigate would be memory. Precisely, it would be relevant to examine the relationship between trauma (e.g., maltreatment), depression, and memory, given the important role of the HPA axis and cortisol in this relationship^35,36,57,69^.

## Conclusion

In conclusion, the results found have great relevance for understanding the association between cumulative experiences of maltreatment and cognitive flexibility as well as the mechanisms in play in this association in order to promote resilience. The findings suggest that one way to reduce the negative association between cumulative experiences of maltreatment over the life course and cognitive flexibility later in life could be the use of interventions targeting and working on diminishing the level of depression. Furthermore, our results highlight the important role of cumulative experiences of maltreatment in childhood in the association between cumulative experiences of maltreatment over the life course and cognitive flexibility. Moreover, to promote resilience after accumulated maltreatment experiences, future research should also determine whether white matter integrity, using DTI, plays a role in the association between cumulative experiences of maltreatment over the life course and performance in cognitive flexibility and determine whether other factors underlie this association.

## Supplementary Information

Supplementary Information.

## Data Availability

The data used in this study came from UK Biobank (http://www.ukbiobank.ac.uk) application number 15697 (PI John Gallacher). Access to the data can be requested through UK Biobank (https://www.ukbiobank.ac.uk/enable-your-research/apply-for-access).
