# Supplementary material for: Cumulative life course adversity, mental health, and cognition in the UK biobank
Source: Sci Rep. 2022 Aug 29;12:14700. doi: 10.1038/s41598-022-18928-9 (PMC9424182; doi:10.1038/s41598-022-18928-9)
Supplement: Supplementary file 1 — Supplementary Information. [file 41598_2022_18928_MOESM1_ESM.docx]

**Figure A**

*Flowchart for participants selection*

**
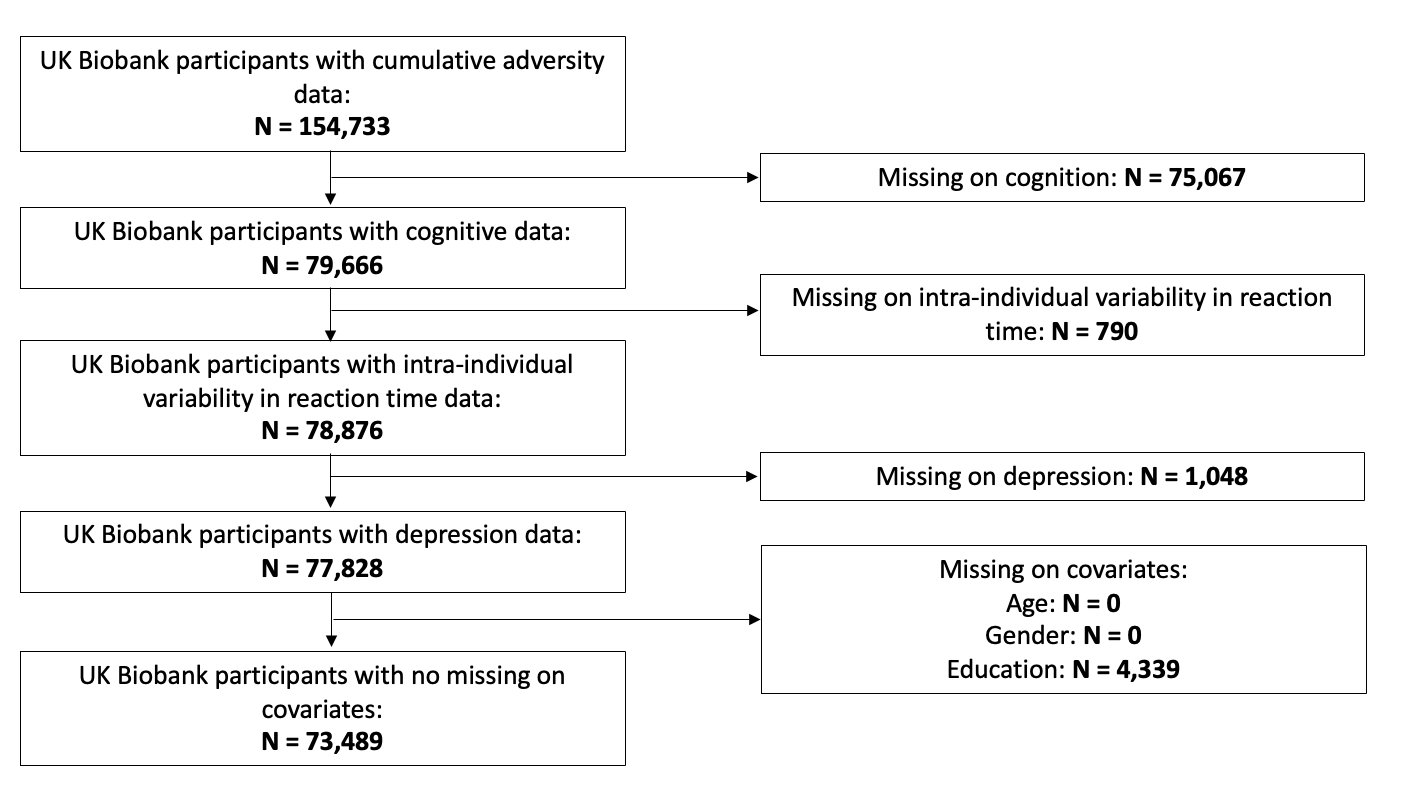
**

**Table A**

*Standardized coefficients of model 1 (cumulative adversity experienced in childhood), with z, p-value, and associated 95% confidence interval*

| Dependent variables | Predictors | Standardized coefficients | *z* | *p*-value | 95% confidence interval | |
| --- | --- | --- | --- | --- | --- | --- |
|  |  |  |  |  | Lower limit | Upper limit |
| TMT ratio |  |  |  |  |  |  |
|  | Childhood life course adversity | .025 | 6.52 | < .001 | .017 | .032 |
|  | CV | .027 | 7.01 | < .001 | .019 | .034 |
|  | Depression | .023 | 5.99 | < .001 | .015 | .030 |
|  | Age | .091 | 24.35 | < .001 | .084 | .098 |
|  | Gender | .017 | 4.55 | < .001 | .010 | .024 |
|  | Education | .045 | 11.96 | < .001 | .038 | .052 |
| CV |  |  |  |  |  |  |
|  | Childhood life course adversity | .004 | 1.05 | .293 | -.003 | .011 |
|  | Age | .074 | 20.54 | < .001 | .067 | .081 |
| Depression |  |  |  |  |  |  |
|  | Childhood life course adversity | .172 | 44.15 | < .001 | .165 | .180 |
|  | Age | -.135 | -37.34 | < .001 | -.142 | -.128 |
|  | Gender | -.059 | -16.37 | < .001 | -.066 | -.052 |

CV = Coefficient of variation.

TMT = Trail Making Test.

*N* = 73,659

CFI = .969

RMSEA = .026

SRMR = .009

**Table B**

*Standardized coefficients of the indirect effect of cumulative adversity experienced in childhood on TMT ratio via CV and depression (Model 1)*

| Mediator | Standardized coefficients | *z* | *p*-value | 95% confidence interval | |
| --- | --- | --- | --- | --- | --- |
|  |  |  |  | Lower limit | Upper limit |
| CV | .001 | 1.04 | .299 | -.001 | .001 |
| Depression | .004 | 5.93 | < .001 | .003 | .005 |

CV = Coefficient of variation.

**Table C**

*Standardized coefficients of model 2 (cumulative adversity experienced in adulthood), with z, p-value, and associated 95% confidence interval*

| Dependent variables | Predictors | Standardized coefficients | *z* | *p*-value | 95% confidence interval | |
| --- | --- | --- | --- | --- | --- | --- |
|  |  |  |  |  | Lower limit | Upper limit |
| TMT ratio |  |  |  |  |  |  |
|  | Adulthood life course adversity | -.001 | -0.21 | .832 | -.008 | .007 |
|  | CV | .027 | 7.07 | < .001 | .019 | .034 |
|  | Depression | .027 | 7.04 | < .001 | .019 | .034 |
|  | Age | .089 | 23.97 | < .001 | .082 | .096 |
|  | Gender | .016 | 4.34 | < .001 | .009 | .023 |
|  | Education | .045 | 11.99 | < .001 | .038 | .052 |
| CV |  |  |  |  |  |  |
|  | Adulthood life course adversity | .010 | 2.57 | .010 | .002 | .017 |
|  | Age | .075 | 20.74 | < .001 | .068 | .082 |
| Depression |  |  |  |  |  |  |
|  | Adulthood life course adversity | .197 | 50.98 | < .001 | .190 | .205 |
|  | Age | -.137 | -38.45 | < .001 | -.144 | -.130 |
|  | Gender | -.027 | -7.60 | < .001 | -.035 | -.020 |

CV = Coefficient of variation.

TMT = Trail Making Test.

*N* = 74,133

CFI = .975

RMSEA = .025

SRMR = .008

**Table D**

*Standardized coefficients of the indirect effect of cumulative adversity experienced in adulthood on TMT ratio via CV and depression (Model 2)*

| Mediator | Standardized coefficients | *z* | *p*-value | 95% confidence interval | |
| --- | --- | --- | --- | --- | --- |
|  |  |  |  | Lower limit | Upper limit |
| CV | .001 | 2.41 | .016 | .001 | .001 |
| Depression | .005 | 6.97 | < .001 | .004 | .007 |

CV = Coefficient of variation.

**Table E**

*Standardized coefficients of model 3 (cumulative adversity experienced in childhood and adulthood), with z, p-value, and associated 95% confidence interval*

| Dependent variables | Predictors | Standardized coefficients | *z* | *p*-value | 95% confidence interval | |
| --- | --- | --- | --- | --- | --- | --- |
|  |  |  |  |  | Lower limit | Upper limit |
| TMT ratio |  |  |  |  |  |  |
|  | Childhood life course adversity | .026 | 6.63 | < .001 | .018 | .033 |
|  | Adulthood life course adversity | -.007 | -1.93 | .053 | -.015 | .001 |
|  | CV | .027 | 7.03 | < .001 | .019 | .034 |
|  | Depression | .023 | 6.03 | < .001 | .016 | .031 |
|  | Age | .092 | 24.48 | < .001 | .084 | .099 |
|  | Gender | .008 | 2.33 | .020 | .001 | .015 |
|  | Education | .045 | 11.94 | < .001 | .038 | .053 |
| CV |  |  |  |  |  |  |
|  | Childhood life course adversity | .002 | .047 | .639 | -.006 | .009 |
|  | Adulthood life course adversity | .010 | 2.46 | .014 | .002 | .017 |
|  | Age | .075 | 20.63 | < .001 | .068 | .082 |
| Depression |  |  |  |  |  |  |
|  | Childhood life course adversity | .135 | 33.41 | < .001 | .128 | .143 |
|  | Adulthood life course adversity | .171 | 41.98 | < .001 | .163 | .179 |
|  | Age | -.128 | -35.56 | < .001 | -.136 | -.121 |
|  | Gender | -.016 | -2.64 | .008 | -.028 | -.004 |

CV = Coefficient of variation.

TMT = Trail Making Test.

*N* = 74,489

CFI = .978

RMSEA = .025

SRMR = .413

**Table F**

*Standardized coefficients of the indirect effect of cumulative adversity experienced in childhood and adulthood on TMT ratio via CV and depression (Model 3)*

| Life course | Mediator | Standardized coefficients | *z* | *p*-value | 95% confidence interval | |
| --- | --- | --- | --- | --- | --- | --- |
|  |  |  |  |  | Lower limit | Upper limit |
| Childhood |  |  |  |  |  |  |
|  | CV | .001 | .047 | .640 | -.001 | .001 |
|  | Depression | .003 | 5.93 | < .001 | .002 | .004 |
| Adulthood |  |  |  |  |  |  |
|  | CV | .001 | 2.31 | .021 | .001 | .001 |
|  | Depression | .004 | 5.97 | < .001 | .003 | .005 |

CV = Coefficient of variation.

**Figure B**

*Simplified illustration of the significant paths found in the models tested*


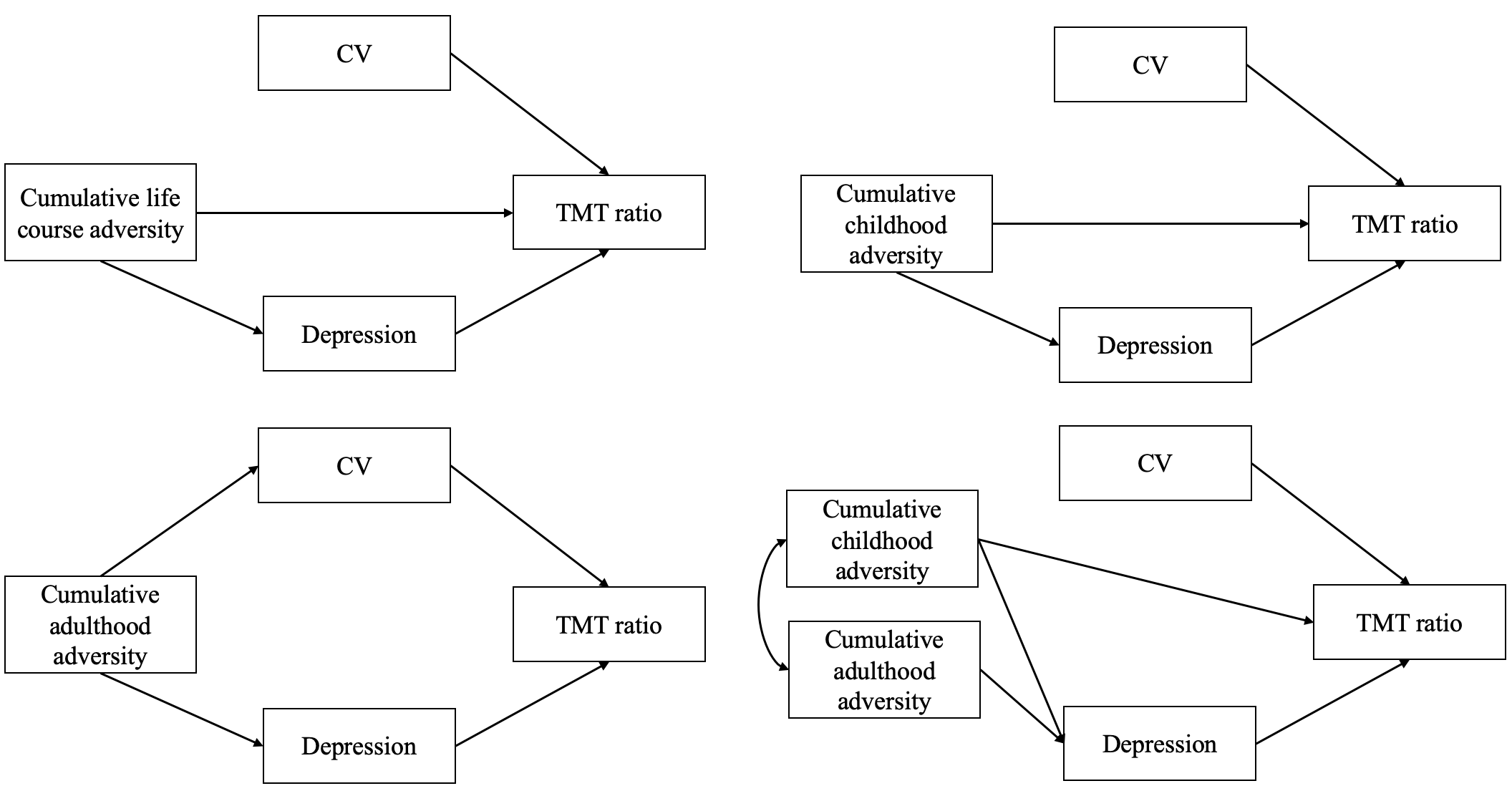


CV = Coefficient of variation.

TMT = Trail Making Test.
